# SERPINB3 enhances NPM1 sumoylation via inhibiting SENP3’s activity and promotes lung tumorigenesis

**DOI:** 10.1038/s41419-025-08347-9

**Published:** 2025-12-24

**Authors:** Xiangyu Meng, Weize Wang, Namratha Sheshadri, Tianyu Zhang, Xinlu Han, Yifu Sun, Man Xiao, Yu Feng, Shan Gao, Jian Chen, Sisi Xie, Yongbo Wang, Yunlong Yang, Yanxiang Zhao, Yuxin Yin, Wei-Xing Zong, Shenglan Gao

**Affiliations:** 1 Department of Cellular and Genetic Medicine, School of Basic Medical Sciences, Fudan University, Shanghai, 200032 China; 2 Institute of Systems Biomedicine, Peking-Tsinghua Center for Life Sciences, School of Basic Medical Sciences, Peking University Health Science Center, Beijing, 100191 China; 3 Department of Chemical Biology, Ernest Mario School of Pharmacy, Rutgers-The State University of New Jersey, Piscataway, NJ 08854 USA; 4 Department of Applied Biology and Chemical Technology, The Hong Kong Polytechnic University, Hung Hom, Kowloon, Hong Kong, China; 5 Department of Thoracic Surgery, Shanghai Pulmonary Hospital, Tongji University, 507 Zhengmin Road, Shanghai, 200433 China; 6 Department of Cardiology, Basic Scientific Research Center, Longyan First Affiliated Hospital of Fujian Medical University, Longyan, 364000 China

**Keywords:** Non-small-cell lung cancer, Oncogenesis, Sumoylation

## Abstract

SERPINB3 or SCCA1, a member of the serine proteinase inhibitor family, is frequently overexpressed in multiple malignancies, including lung adenocarcinoma (LUAD). However, its molecular mechanisms and endogenous substrates in lung cancer remain poorly characterized. Here, we identified SUMO-specific proteinase 3 (SENP3) as a novel direct interaction partner and functional target of SERPINB3 in LUAD. Using proximity-dependent biotin labeling coupled with mass spectrometry, we discovered that SERPINB3 forms a nuclear complex with SENP3 and nucleophosmin (NPM1). This tripartite interaction was validated through endogenous co-immunoprecipitation and bimolecular fluorescence complementation assays. Structural modeling with AlphaFold3 and subsequent mutagenesis studies revealed that SERPINB3 binds specifically to the N-terminal region of SENP3 (amino acids 265-287). Furthermore, our structural and biochemical analyses demonstrate that SENP3 specifically interacts with the C-terminal aromatic domain of NPM1 (amino acids 242-294), while SERPINB3 selectively binds to the pentameric form of NPM1. Mechanistically, SERPINB3 inhibits SENP3’s desumoylation activity, leading to enhanced NPM1 sumoylation in the nucleolus. Functional studies demonstrated that SERPINB3 overexpression (but not the RCL-deleted mutant, SERPINB3△6) promotes LUAD cell proliferation and tumor growth, with NPM1 sumoylation being critical for this oncogenic effect. Clinically, SERPINB3 is highly expressed in human LUAD specimens and co-localizes with SENP3 and NPM1. Our work establishes SENP3 as a bona fide endogenous substrate of SERPINB3 and delineates a novel sumoylation-dependent oncogenic axis in LUAD, offering potential therapeutic targets for this malignancy.

## Introduction

Serine Proteinase Inhibitor clade B member 3 (SERPINB3), or Squamous Cell Carcinoma Antigen 1 (SCCA1), was initially identified as being overexpressed in squamous cell carcinoma (SCC) of the uterine cervix [1]. Subsequent studies have revealed its upregulation in a wide range of malignancies, including lung [2, 3], head and neck [4], hepatocellular carcinoma [5], pancreatic ductal adenocarcinoma [6], and breast cancer [7, 8].

SERPINB3 is a member of the ovalbumin serpin family and functions as a cross-class inhibitor [9], targeting cysteine proteinases such as the papain-like lysosomal cathepsins K, L, and S [9, 10]. It exerts its inhibitory activity via a “bait-and-trap” pseudosubstrate mechanism, whereby its carboxyl-terminal reactive center loop (RCL) inserts into the active site of the target protease, leading to irreversible inhibition [9, 11–14].

Traditionally, SERPINB3 has been regarded as a secretory protein released by tumor cells and detected in patient serum as a biomarker [15]. Recent studies have explored its multifaceted roles in cancer development [16]. Beyond its involvement in epithelial-mesenchymal transition (EMT) and tumor metastasis [6, 17], SERPINB3 contributes to therapeutic resistance, including chemo-, radio-, and immunotherapy [18–21]. Depending on its subcellular localization, SERPINB3 has different functions. In the cytosol, SERPINB3 inhibits lysosomal injury-induced cell death by suppressing cathepsin L release, thereby protecting cells from lysoptosis [18]. Emerging evidence indicates that SERPINB3 can also translocate to the nucleus, where it exerts distinct regulatory functions [22, 23]. For example, nuclear SERPINB3 interacts with phosphorylated JNK, attenuating its kinase activity and conferring resistance to UV-induced apoptosis [23]. Nevertheless, the precise mechanisms underlying SERPINB3’s nuclear functions remain incompletely understood and warrant further investigation.

Non-small cell lung carcinoma (NSCLC) represents the most common and deadly form of lung cancer worldwide, accounting for approximately 85% of all lung cancer cases [24, 25]. This heterogeneous disease is primarily classified into two major subtypes: lung adenocarcinoma (LUAD, ~40%) and lung squamous cell carcinoma (LUSC, ~25%) [24]. Notably, elevated serum levels of SERPINB3 have been observed in both LUAD and LUSC patients [2, 26], suggesting its potential role in NSCLC pathogenesis. Supporting this notion, transgenic mouse models demonstrate that SERPINB3 overexpression drives epithelial proliferation and induces pulmonary fibrosis [3]. Despite these clinical and preclinical observations, the intracellular targets of SERPINB3 and the precise molecular mechanisms through which it promotes lung tumorigenesis remain poorly understood.

Sumoylation, a reversible post-translational modification involving the covalent attachment of small ubiquitin-like modifier (SUMO) proteins, plays a critical role in regulating diverse cellular processes [27–29]. This modification cascade requires sequential action of E1 activating enzymes, the E2 conjugating enzyme UBC9, and often E3 ligases for substrate specificity. Conversely, SUMO deconjugation is mediated by sentrin-specific proteases (SENPs), which maintain sumoylation homeostasis [30]. Growing evidence implicates aberrant sumoylation in tumorigenesis, with elevated SUMO modification observed across multiple cancer types [29]. Notably, pharmacological inhibition of the SUMO pathway has emerged as a promising therapeutic strategy, particularly in pancreatic cancer [31, 32]. Despite these advances, the functional significance and regulatory mechanisms of sumoylation in NSCLC remain poorly understood.

In this study, we attempted to identify novel molecular interactors of SERPINB3 in LUAD cells through proteomic analysis. Our results demonstrate that SERPINB3 forms a complex with SENP3 and NPM1 within the nucleolar compartment. Mechanistically, SERPINB3 functions as a potent inhibitor of SENP3’s desumoylation activity, leading to global hyper-sumoylation and specific accumulation of sumoylated NPM1. Furthermore, this SERPINB3-SENP3-NPM1 axis significantly promotes LUAD cell proliferation, revealing a previously unrecognized oncogenic mechanism in lung adenocarcinoma pathogenesis.

## Results

### SERPINB3 is overexpressed in human LUAD and LUSC and associates with poor prognosis in LUAD

**Fig. 1:**
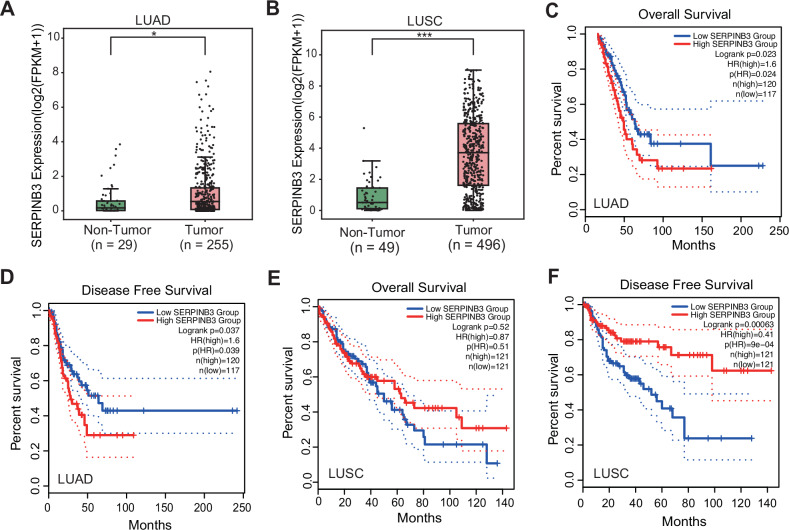
SERPINB3 is overexpressed in human LUAD and LUSC and associates with poor prognosis in LUAD. Box plots comparison of the SERPINB3 expression levels between non-tumor (NT) and tumor (T) tissues of LUAD samples (**A**) and LUSC samples (**B**) from the TCGA database. TCGA: The Cancer Genome Atlas, FPKM: fragments per kilobase of transcript per million fragments. The statistical significance was determined by Wilcoxon rank-sum test. *p < 0.05, ***p < 0.001. Kaplan-Meier survival curves of overall survival (**C**) and disease-free survival (**D**) for LUAD patients from the TCGA cohort stratified by upper quartile (high) and lower quartile (low) of SERPINB3 expression levels. The statistical significance was determined by log-rank test. *p < 0.05. Kaplan-Meier survival curves of overall survival (**E**) (n.s.: no significance) and disease-free survival (**F**) (***p < 0.001) for LUSC patients from the TCGA cohort stratified by upper quartile (high) and lower quartile (low) of SERPINB3 expression levels. The statistical significance was determined by log-rank test.

To elucidate the role of SERPINB3 in lung tumorigenesis, we analyzed transcriptomic data from The Cancer Genome Atlas (TCGA) and observed significant upregulation of SERPINB3 in both LUAD and LUSC patients (Fig. 1A, B). Elevated SERPINB3 expression was associated with reduced overall survival (OS) and disease-free survival (DFS) in LUAD patients, as well as supporting its potential pro-tumorigenic function in this subtype (Fig. 1C, D). Intriguingly, despite its high expression in LUSC (Fig. 1B), SERPINB3 levels correlated with improved OS and DFS in LUSC patients (Fig. 1E, F), suggesting a subtype-specific regulatory role of SERPINB3 in lung cancer progression. The prognostic significance of SERPINB3 remained consistent when analyses were stratified by disease stage (Fig. S1A-H). Given these divergent associations, we focused our subsequent investigations on dissecting the mechanistic contributions of SERPINB3 to LUAD tumorigenesis.

### Identification of SENP3 and NPM1 as novel SERPINB3-interacting proteins

**Fig. 2:**
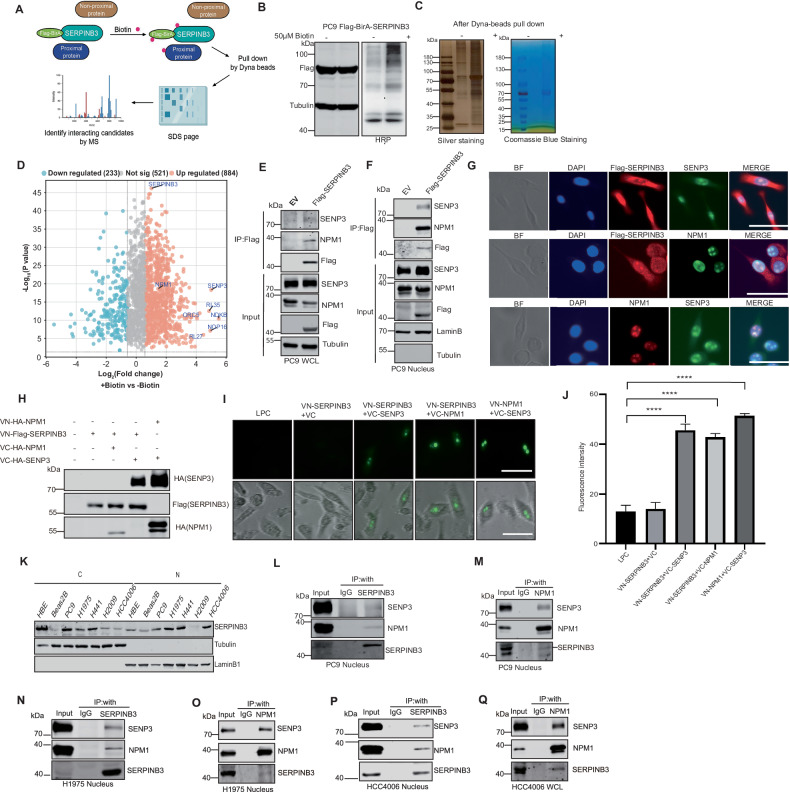
Identification of SENP3 and NPM1 as novel SERPINB3-interacting proteins. **A** Diagram summarizing the main procedures of Bio-ID. **B** Western blot analysis and HRP staining of PC9 cells overexpressing Flag-BirA-SERPINB3 treated with or without 50 μM biotin for 24 h. **C** Silver staining and Coomassie blue staining of protein lysates from (**B**) after Dynabeads pull down. **D** The volcano plot shows SERPINB3’s interacting proteins after mass spectrometry analysis. Cutoffs: Fold change >1.5, *p* < 0.05. Western blot analysis of SENP3 and NPM1 after immunoprecipitated with Flag antibody in whole lysates (**E**) or nuclear fractions (**F**) of PC9 cells overexpressing Flag-SERPINB3. EV empty vector. **G** Co-IF of Flag, SENP3 and NPM1 in PC9 cells overexpressing Flag-SERPINB3. BF: bright field. Scale bars: 50 μm. **H** Western blot analysis of proteins from PC9 cells transfected with various VN and VC constructs. **I** Representative fluorescent images of PC9 cells transfected with indicated VN and VC constructs. Scale Bars: 50 μm. **J** Quantification of fluorescent intensity of PC9 cells transfected with indicated VN and VC constructs. Values are mean ± SD of three countings, ****p < 0.0001. The statistical significance was determined by the Student’s unpaired *t*-test. **K** Western blot analysis of cytoplasmic (C) and nuclear (N) SERPINB3 in various lung cell lines. α-Tubulin and LaminB1 serve as loading control. Western blot analysis of SENP3, NPM1 and SERPINB3 after immunoprecipitated with endogenous SERPINB3 (**L**) or with endogenous NPM1 (**M**) in PC9 nuclear lysates. IgG serves as IP control. Western blot analysis of SENP3, NPM1 and SERPINB3 after immunoprecipitated with endogenous SERPINB3 (**N**) or endogenous NPM1 (**O**) in H1975 nuclear lysates. IgG serves as IP control. Western blot analysis of SENP3, NPM1 and SERPINB3 after immunoprecipitated with endogenous SERPINB3 (**P**) or endogenous NPM1 (**Q**) in HCC4006 nuclear lysates. IgG serves as IP control.

To elucidate the molecular mechanisms underlying SERPINB3’s role in LUAD tumorigenesis, we employed proximity-dependent biotin identification (Bio-ID) to map SERPINB3-interacting proteins in PC9 LUAD cells under physiological conditions (Fig. 2A). Bio-ID leverages BirA*, a mutant *Escherichia coli* biotin ligase that promiscuously biotinylates proximal proteins [33]. Stable PC9 cell lines expressing Flag-BirA-SERPINB3 exhibited enhanced biotinylation activity (Fig. 2B). Biotinylated proteins were affinity-purified using streptavidin Dynabeads and validated by silver staining (Fig. 2C). Following Coomassie blue staining (Fig. 2C), mass spectrometry (MS) analysis identified SERPINB3 as a predominant hit, with the cysteine protease SENP3 and its nuclear target NPM1 among the top-scoring interactors (Fig. 2D). Co-immunoprecipitation (Co-IP) of Flag-SERPINB3 from PC9 lysates confirmed these interactions (Fig. 2E). SENP3, a nucleolar protein critical for nuclear sumoylation homeostasis, regulates NPM1 sumoylation [34]. Consistent with this, SERPINB3-SENP3-NPM1 complexes were enriched in nuclear fractions (Fig. 2F). Co-immunofluorescence (Co-IF) using anti-Flag, anti-SENP3, and anti-NPM1 antibodies further demonstrated nuclear co-localization of ectopically expressed SERPINB3 with endogenous SENP3 and NPM1 (Fig. 2G). Although SERPINB3 localized to both nucleus and cytosol, these data highlight its nuclear association with SENP3 and NPM1, suggesting a functional interplay in this compartment.

To further characterize the protein-protein interactions, we employed bimolecular fluorescence complementation (BiFC) analysis [35]. SERPINB3, SENP3, and NPM1 were individually fused to the N-terminal (VN, residues 1-173) or C-terminal (VC, residues 155-239) fragments of Venus fluorescent protein and expressed in PC9 cells (Fig. 2H). Distinct fluorescence complementation was observed in cells co-expressing VN-SERPINB3 with VC-SENP3 (Fig. 2I) or VN-SERPINB3 with VC-NPM1 (Fig. 2J), demonstrating direct interaction between these protein pairs. As a positive control, co-expression of VN-NPM1 with VC-SENP3 similarly produced fluorescent signals (Fig. 2I, J). These BiFC results provide visual confirmation of specific interactions between SERPINB3 and both SENP3 and NPM1 in living cells.

Moreover, at the endogenous level, SERPINB3 was detected in both the cytosol and nucleus across multiple lung cell lines and LUAD cells (Fig. 2K). However, nuclear SERPINB3 was not detected by western blot in LUSC cell lines (Fig. S2). Importantly, co-immunoprecipitation (Co-IP) assays demonstrated interactions between SERPINB3 and both SENP3 and NPM1 in the nucleus in PC9 (Fig. 2L, M), H1975 (Fig. 2N, O) and HCC4006 cells (Fig. 2P, Q). Together, these findings demonstrate that SERPINB3 forms nuclear complexes with SENP3 and NPM1 in LUAD cells.

### SERPINB3 forms a protein complex with SENP3 and NPM1

**Fig. 3:**
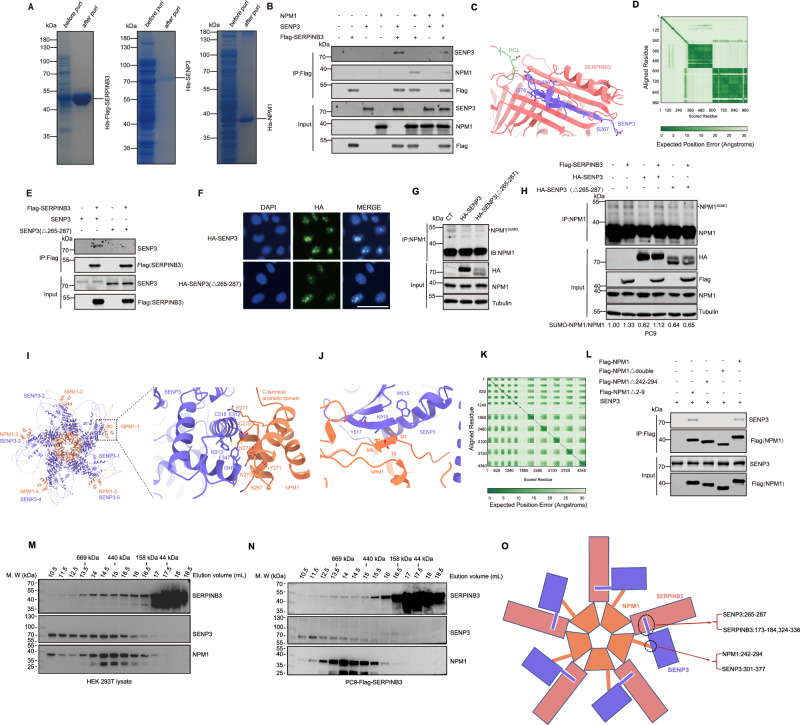
SERPINB3 forms a protein complex with SENP3 and NPM1. **A** Coomassie blue staining of SERPINB3, SENP3 and NPM1 proteins before and after purification from *E-coli*. **B** Western blot analysis of SENP3 and NPM1 after immunoprecipitated with Flag antibody using SENP3, NPM1 and Flag-SERPINB3 purified proteins. Experiments were repeated twice. **C** Cartoon model of the SENP3-SCCA1 complex predicted by AlphaFold3. **D** Predicted aligned error (PAE) map of the SENP3-SCCA1 complex predicted by AlphaFold3. **E** Western blot analysis of SENP3 and truncated SENP3 (Δ265-287) after Co-IP using Flag antibody with purified proteins. **F** Immunofluorescent analysis of HA tag in PC9 cells overexpressing wild-type SENP3 and SENP3 Δ265-287. Scale bars: 50 μm. **G** Western blot analysis of NPM1 sumoylation after immunoprecipitated with NPM1 antibody in PC9 cells overexpressing wild-type SENP3 and SENP3 Δ265-287. **H** Western blot analysis of NPM1 sumoylation after immunocipitated with NPM1 antibody in PC9 cells transfected with Flag-SERPINB3, HA-SENP3 and HA-SENP3(△265-287) viruses as indicated. Experiments were repeated twice. The ratios of SUMO2-NPM1/NPM1 were calculated and indicated. **I**, **J** Cartoon model of the pentameric NPM1-SENP3 complex predicted by AlphaFold3. **K** Predicted aligned error (PAE) map of the pentameric NPM1-SENP3 complex predicted by AlphaFold3. **L** Western blot analysis of SENP3 after Co-IP using Flag antibody with purified proteins. **M** Lysates from HEK293 cells transfected with plasmids encoding Flag-SERPINB3, HA-NPM1, and His-SENP3 were separated on a Superose 6 Increase 10/300 column. Fractions were resolved by SDS-PAGE and probed with the indicated antibodies. **N** Lysates from PC9 cells overexpressing Flag-SERPINB3 were separated on a Superose 6 Increase 10/300 column. Fractions were resolved by SDS-PAGE and probed with the indicated antibodies. **O** Proposed model of SERPINB3/SENP3/NPM1 complex.

To determine whether the interactions between SERPINB3, SENP3, and NPM1 are direct, we expressed and purified recombinant proteins in E. coli (Fig. 3A). In vitro immunoprecipitation assays confirmed reciprocal interactions among the three proteins (Fig. 3B). Leveraging recent advances in artificial intelligence-based protein structure prediction [36, 37], we employed AlphaFold3 to model these interactions. The predicted SERPINB3-SENP3 complex revealed that the N-terminal disordered region (aa 265-287) of SENP3 forms a β-strand that integrates into SERPINB3’s five-stranded β-sheet structure (Fig. 3C and D), consistent with known stabilizing conformations of SERPINB3 [11, 13]. To validate this prediction, we generated and purified a SENP3 Δ265-287 deletion mutant. Notably, this mutant failed to interact with SERPINB3 (Fig. 3E). Furthermore, the SENP3 Δ265-287 mutant maintained nuclear localization (Fig. 3F) and desumoylase activity comparable to wild-type SENP3 (Fig. 3G). However, this mutant was resistant to the inhibition by SERPINB3 (Fig. 3H), indicating that the 265-287 region is essential for mediating the SERPINB3-SENP3 interaction and the consequent suppression of desumoylation.

While AlphaFold 3 failed to predict a direct interaction between SERPINB3 and NPM1, our co-immunoprecipitation assay indicated they directly interacted (Fig. 3B). Given that full-length NPM1 oligomerizes into pentameric structures that serve as nucleolar scaffolds for protein interactions [38, 39], it is possible that NPM1’s pentameric conformation is essential for SERPINB3 binding. To test this hypothesis, we generated an N-terminal truncation mutant (NPM1Δ1-113) that is incapable of pentamer formation. Immunofluorescent analysis revealed that NPM1Δ1-113 failed to localize to the nucleolus (Fig. S3A-B). Immunoprecipitation analysis demonstrated that NPM1Δ1-113 does not interact with SERPINB3 (Fig. S3C), indicating that SERPINB3-NPM1 interaction indeed depends on NPM1’s oligomeric state.

Structural and biochemical studies have established that NPM1 forms pentameric complexes capable of interacting with SENP3 [34, 39, 40]. AlphaFold3 modeling of the NPM1-SENP3 complex predicted two potential interaction interfaces: the C-terminal aromatic domain (aa 242-294) and N-terminal region (aa 2-9) of NPM1 (Fig. 3I-K). To experimentally validate these predictions, we generated a series of NPM1 truncation mutants: C-terminal deletion (NPM1Δ242-294), N-terminal deletion (NPM1Δ2-9), and a double mutant lacking both domains (NPM1Δdouble). Co-immunoprecipitation analysis demonstrated that the C-terminal, but not N-terminal, truncation significantly disrupted NPM1-SENP3 binding (Fig. 3L). Our data suggest that NPM1-SENP3 complex formation is principally mediated by the C-terminal aromatic domain of NPM1.

To examine whether the SERPINB3-NPM1-SENP3 complex exists in cells, we performed gel filtration of lysates with HEK293T cells containing exogenously expressed Flag-SERPINB3, HA-NPM1, and His-SENP3. We found that all components of the SERPINB3-NPM1-SENP3 complex were eluted at a high molecular weight (>440 kDa), suggesting that the complex exists in cells (Fig. 3M). We further enriched His-tagged SENP3 using Ni-NTA beads and performed gel filtration analysis. The results again showed that all components of the SERPINB3-NPM1-SENP3 complex were eluted at >440 kDa (Fig. S4), consistent with our immunoprecipitation assay showing that SERPINB3 interacts with SENP3 and NPM1. Similar results were also observed in PC9 cells overexpression SERPINB3 (Fig. 3N). Taken together, these results suggest that the SERPINB3-NPM1-SENP3 complex exists in cells.

Based on these results, we propose a molecular model delineating the interactions between SERPINB3, SENP3, and NPM1. Our data demonstrate that SERPINB3 directly binds to the 265-287 amino acid region of SENP3. Furthermore, NPM1 forms a pentameric complex that interacts with SENP3 through its C-terminal aromatic domain (amino acids 242-294). Importantly, we identified a direct association between SERPINB3 and the NPM1 pentameric complex (Fig. 3O). Collectively, these results provide mechanistic insights into the molecular interactions among SERPINB3, SENP3, and NPM1.

### SERPINB3 inhibits iso-peptidase activity of SENP3 and increases NPM1 sumoylation in LUAD cells

**Fig. 4:**
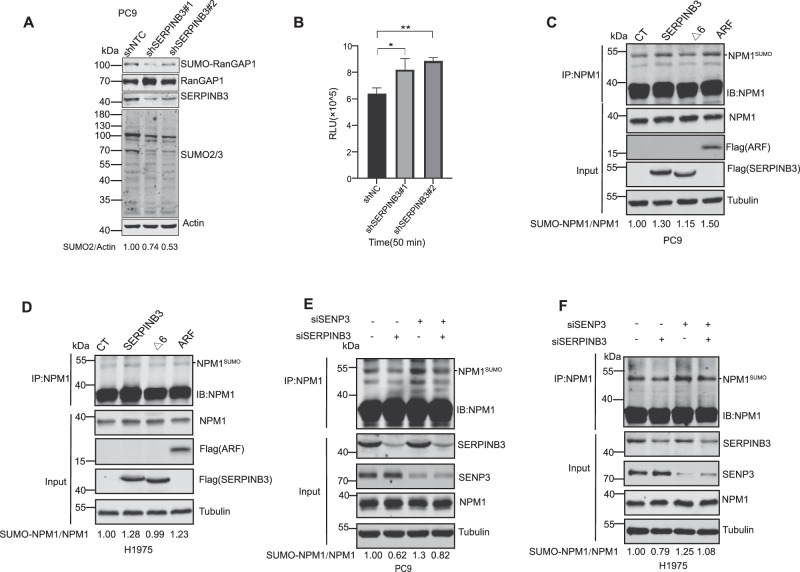
SERPINB3 inhibits iso-peptidase activity of SENP3 and increases NPM1 sumoylation in LUAD cells. **A** Western blot analysis of indicated proteins from control and SERPINB3 knockdown PC9 cells. β-actin serves as loading control. Experiments were repeated twice. The ratio of SUMO2/β-actin was calculated and indicated. **B** Bar graph shows SENP3 activity in control and SERPINB3 knockdown PC9 cells after incubating with hSUMO2-AMC for 50 min. RLU: relative unit. Values are mean ± SD from three independent experiments, *p < 0.05; **p < 0.01. Student’s unpaired *t*-test was utilized to determine the statistical significance. Western blot analysis of NPM1 sumoylation after immunoprecipitated with NPM1 antibody in PC9 (**C**) and H1975 (**D**) control, Flag-SERPINB3 overexpression, Flag-SERPINB3 ∆6 overexpression and Flag-Arf overexpression cells. Experiments for each cell line were repeated twice. The ratios of SUMO2-NPM1/NPM1 were calculated and indicated. Western blot analysis of NPM1 sumoylation after immunoprecipitated with NPM1 antibody in PC9 (**E**) and H1975 (**F**) cells transfected with siRNAs targeting SERPINB3, SENP3 and both, as indicated. Transfection of a scrambled oligonucleotide served as a control. Experiments for each cell line were repeated twice. The ratios of SUMO2-NPM1/NPM1 were calculated and indicated.

SENP3, a cysteine proteinase with a C48 peptidase domain, exhibits both endopeptidase and isopeptidase activities critical for processing SUMO2/3 precursors and deconjugating SUMO2/3 from modified substrates [29, 41]. The result that SERPINB3 directly interacts with SENP3 interaction suggests that SERPINB3 may regulate SENP3’s protease activity. Initial experiments revealed that SERPINB3 knockdown in PC9 cells reduced both SUMO2-RanGAP1 conjugation and global SUMO2 levels, implicating SERPINB3 in SUMOylation homeostasis (Fig. 4A). In addition, we employed a fluorogenic SUMO2-AMC isopeptidase assay [42] to evaluate the SUMO2 deconjugation after SERPINB3 knockdown. In this system, cleavage of the SUMO2-AMC conjugate (where mature SUMO2’s C-terminal glycine is linked to 7-amino-4-methylcoumarin) by desumoylases releases free AMC, detectable by fluorescence emission at 460 nm. Notably, SERPINB3 knockdown increased AMC fluorescence intensity (Fig.4B), demonstrating that SERPINB3 inhibits SUMO2 desumoylation and increases SUMO2 level in the cell. These findings establish SERPINB3 as a novel inhibitor of SUMO2/3 deconjugation.

NPM1, a multifunctional nucleolar phosphoprotein, is SENP3’s direct substrate [34]. Given that SERPINB3, SENP3, and NPM1 form a complex, we sought to examine the effect of SERPINB3 on NPM1 sumoylation. Tumor suppressor Arf (Alternative Reading Frame; p14^ARF^ in human) is a known factor that facilitates NPM1 sumoylation [43, 44]. We generated PC9 cells that stably express wild-type SERPINB3, the RCL-deletion mutant (SERPINB3Δ6) or the Arf. Immunoprecipitation of NPM1 followed by NPM1 immunoblotting revealed that wild-type SERPINB3, as well as Arf, but not SERPINB3Δ6, elevated NPM1 monoSUMOylation (~50 kDa) (Fig. 4C). This effect was conserved in H1975 (Fig. 4D) cell, confirming the RCL-dependent enhancement of NPM1 sumoylation by SERPINB3 in LUAD models.

To delineate the mechanistic relationship between SERPINB3 and SENP3 in regulating NPM1 sumoylation, we performed SERPINB3 and SENP3 silencing, singly or in combination, in PC9 (Fig. 4E) and H1975 (Fig. 4F) cells via siRNA. Our results showed that knockdown of SERPINB3 decreased the sumoylation of NPM1. As predicted, SENP3 depletion increases NPM1 sumoylation, consistent with its established desumoylase function [34]. Intriguingly, dual knockdown of SERPINB3 and SENP3 attenuated NPM1 sumoylation relative to SENP3 depletion alone, demonstrating that SERPINB3 potentiates NPM1 SUMOylation by antagonizing SENP3 activity in an RCL-dependent manner.

### SERPINB3 promotes LUAD cell proliferation and tumorigenesis

**Fig. 5:**
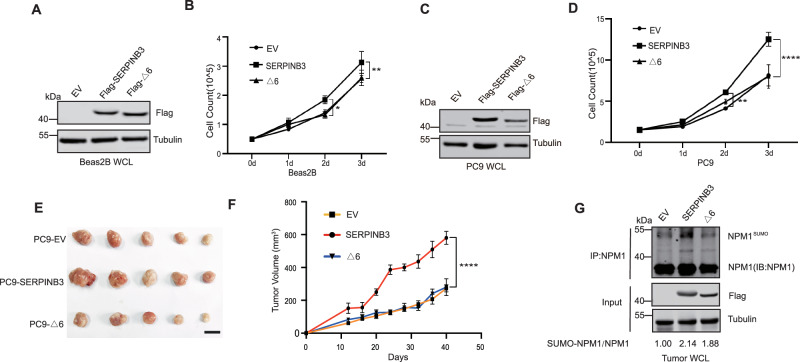
SERPINB3 promotes LUAD cell proliferation and tumorigenesis. **A** Western blot analysis of SERPINB3 expression in Beas2B cells. **B** Growth curve of Beas2B control and SERPINB3 overexpression cells. Seed 5 × 10^4^ Beas2B cells and count for 3 days. Values are mean ± SD from three independent experiments, *p < 0.05, **p < 0.01. Two way ANOVA test was utilized for multiple group comparison. **C** Western blot analysis of SERPINB3 expression in PC9 cells. **D** Growth curve of Beas2B control and SERPINB3 overexpression cells. Seed 1.5 × 10^5^ PC9 cells and count for 3 days. Values are mean ± SD from three independent experiments, **p < 0.01, ****p < 0.0001. Two way ANOVA test was utilized for multiple group comparison. **E** Xenograft tumors growing from indicated PC9 cell lines were isolated from nude mice after inoculation for 40 days. **F** Xenograft tumor volume curve of PC9 cells overexpressing empty vector (EV), SERPINB3 and △6. Values are mean ± SEM, *N* = 5 mice for each group, ****p < 0.0001. Two way ANOVA test was utilized for multiple group comparison. **G** Western blot analysis of NPM1 sumoylation after immunoprecipitated with NPM1 antibody from xenograft tumor lysates. The ratio of SUMO2-NPM1/NPM1 was indicated.

To elucidate the oncogenic potential of SERPINB3 in lung tumorigenesis, we first evaluated its growth-promoting effects in the immortalized yet untransformed lung bronchial epithelial cells (Beas2B). Ectopic expression of wild-type SERPINB3, but not the RCL-deletion mutant (SERPINB3△6) significantly enhanced Beas2B proliferation (Fig. 5A, B). This enhanced proliferation upon SERPINB3 expression also occurred in LUAD PC9 cells (Fig. 5C, D). Tumor xenograft studies further confirmed that wild-type SERPINB3, but not SERPINB3△6, robustly promoted tumor growth (Fig. 5E, F), which was accompanied by elevated NPM1 sumoylation in SERPINB3-overexpressing tumors compared to both control and SERPINB3△6 groups (Fig. 5G). Collectively, these results demonstrate that SERPINB3 drives lung tumor cell proliferation and tumorigenesis in an RCL-dependent manner, and are consistent with the theory that increased NPM1 sumoylation is involved in this process.

### NPM1 sumoylation is critical for its tumor-promoting function in LUAD

**Fig. 6:**
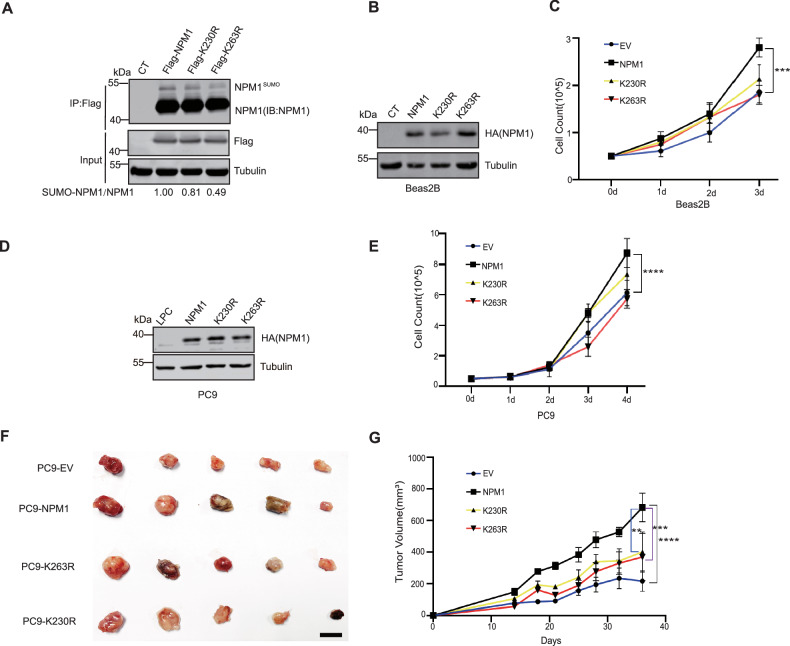
NPM1 sumoylation is critical for its tumor-promoting function in LUAD. **A** Western blot analysis of NPM1 and NPM1 mutants (K230R and K263R) sumoylation in PC9 cells. The ratio of SUMO2-NPM1/tubulin was indicated. **B** Western blot analysis of NPM1 and NPM1 mutant (K230R and K263R) expression in Beas2B cells. **C** Growth curve of Beas2B control, NPM1 and NPM1 mutants (K230R and K263R) overexpression cells. Seed 5×10^4^ Beas2B cells and count for 3 days. Values are mean ± SD from three independent experiments, ***p < 0.001. Two way ANOVA test was utilized for multiple group comparison. **D** Western blot analysis of NPM1 and NPM1 mutants (K230R and K263R) expression in PC9 cells. **E** Growth curve of PC9 control, NPM1 and NPM1 mutants (K230R and K263R) overexpression cells. Seed 1.5×10^5^ PC9 cells and count for 3 days. Values are mean ± SD from three independent experiments, ****p < 0.0001. Two way ANOVA test was utilized for multiple group comparison. **F** Xenograft tumors growing from indicated PC9 cell lines were isolated from nude mice after inoculation for 36 days. **G** Xenograft tumor volume curve of PC9 control, NPM1 and NPM1 mutants (K230R and K263R) overexpression cells. Values are mean ± SEM, *N* = 5 mice for each group, **p < 0.01, ***p < 0.001, ****p < 0.0001. Two way ANOVA test was utilized for multiple group comparison.

NPM1 sumoylation has been suggested to regulate cell proliferation, where K230 and K263 were identified as the sumoylation sites, with K263 being the major modification site [43]. To investigate the functional significance of NPM1 sumoylation in lung cancer, we generated PC9 cells stably expressing wild-type NPM1 or sumoylation-impaired mutants (K230R and K263R). Immunoprecipitation analysis confirmed significantly reduced sumoylation of both mutants compared to wild-type NPM1 (Fig. 6A). In addition, the NPM1 K230/263 R double mutant exhibited a more pronounced reduction in sumoylation level compared to wild-type NPM1 (Fig. S5A).

We next examined the biological consequences of these modifications by overexpressing wild-type and mutant NPM1 in Beas2B cells (Fig. 6B). Both K230R and K263R mutants exhibited impaired proliferative capacity relative to wild-type NPM1, with the K263R mutation showing more profound effects (Fig. 6C). Similar effect was observed in the LUAD PC9 cells, where wild-type NPM1 enhanced proliferation while the sumoylation-deficient mutants did not (Fig. 6D, E). Consistently, the NPM1 K230/263 R double mutant exhibited a more pronounced impairment of cell proliferation compared to the wild-type protein (Fig. S5B-E). In vivo xenograft studies further demonstrated that tumors expressing K230R or K263R mutants displayed significantly reduced growth compared to wild-type NPM1-expressing tumors (Fig. 6F, G). These results indicate that NPM1 sumoylation promotes tumor cell growth in LAUD.

### SERPINB3 is upregulated in human LUAD samples and co-localizes with SENP3 and NPM1

**Fig. 7:**
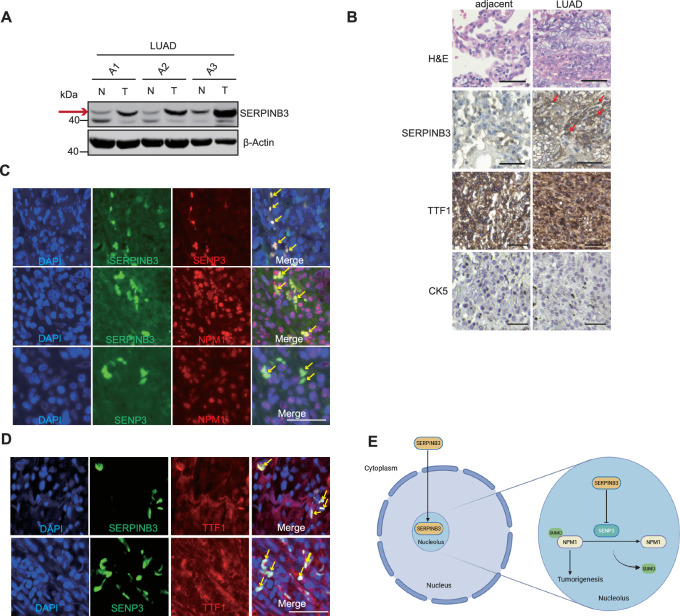
SERPINB3 is upregulated in human LUAD samples and co-localizes with SENP3 and NPM1. **A** Western blot analysis of SERPINB3 from matched adjacent and human LUAD tumor samples. N: tumor adjacent tissue. T: tumor sample. β-actin serves as loading control. Red arrow indicates the band of SERPINB3. **B** Representative images of Hematoxylin and Eosin (H&E) staining of matched adjacent and human LUAD samples. Representative images of immunohistochemistry results of SERPINB3, TTF1 and CK5 were shown. Scale Bars: 50 μm. Red arrows indicate nuclear staining of SERPINB3. **C** Representative images of immunofluorescent staining of DAPI, SERPINB3, SENP3 and NPM1 in human LUAD samples were shown. Scale Bars: 50 μm. Yellow arrows indicate overlapping of immunofluorescent signals. **D** Representative images of immunofluorescent staining of DAPI, SERPINB3, SENP3 and TTF1 in human LUAD samples were shown. Scale Bars: 50 μm. Yellow arrows indicate overlapping of immunofluorescent signals. **E** Graphic image showing the central finding of the study: Nuclear SERPINB3 forms complex with SENP3 and NPM1, inhibits SENP3’s desumoylation activity, enhances NPM1 sumoylation and contributes to tumorigenesis in LUAD. The image was created in https://BioRender.com.

To assess SERPINB3 expression patterns in clinical LUAD specimens, we analyzed matched pairs of tumor and adjacent normal tissues. Western blot analysis showed markedly elevated SERPINB3 protein levels in tumor tissues compared to the adjacent normal tissues (Fig. 7A). Immunohistochemical staining also showed elevated SERPINB3 expression in LUAD samples, with both cytoplasmic and nuclear localization (Fig. 7B). Histopathological characterization using established markers revealed positive staining for thyroid transcription factor 1 (TTF1, a LUAD marker) and negative staining for cytokeratin 5 (CK5, a LUSC marker), confirming the adenocarcinoma phenotype of these samples (Fig. 7B). Immunofluorescence analysis of clinical specimens revealed significant co-localization of SERPINB3 with both SENP3 and NPM1 (Fig. 7C), mirroring our in vitro observations in cell culture (Fig. 2G). Furthermore, SERPINB3 and SENP3 are expressed in tumor cells as confirmed by co-staining with the lung adenocarcinoma marker TTF1 (Fig.7D).

## Discussion

Our study reveals a novel mechanism by which SERPINB3, through its dynamic tertiary structure to trap proteinases [10], interacts with and inhibits the cysteine protease SENP3 in LUAD cells. We demonstrate that both ectopically expressed and endogenous SERPINB3 form a complex with SENP3 and NPM1, regulating NPM1 sumoylation status. Given that NPM1 sumoylation plays critical roles in tumorigenesis, our finding suggests that SERPINB3 can promote malignant proliferation of LUAD by regulating SENP3-mediated NPM1 sumoylation (Fig. 7E).

In our study, we demonstrated for the first time that SENP3’s N-terminal disordered region (residues 265-287) is a prerequisite for the inhibition of SENP3’s desumoylation activity. Based on our findings, we propose a model in which the disordered region of SENP3 mediates the initial recruitment of SERPINB3, which facilitates the formation of the Michaelis encounter complex with SERPINB3, allowing SERPINB3 to employ its reactive center loop (RCL) to inhibit SENP3’s catalytic domain. Actually, this “bind-then-inhibit” mechanism is a common and efficient strategy in biology. The initial interaction ensures specificity, and the subsequent inhibition takes place. Therefore, our findings provide additional information to the “bait-and-trap” inhibition mechanism for SERPINs’ interaction with its substrates.

Emerging evidence indicates that SERPINB3 possesses oncogenic functions beyond its canonical cytoplasmic roles. A recent glioblastoma study revealed that nuclear-localized SERPINB3 may contribute to cancer stem cell maintenance [45]. Meanwhile, it has been shown that nuclear SERPINB3 can interact with USP1 and promotes FANCD2-FANCI ubiquitination, enhances DNA damage repair capacity and decreases cisplatin sensitivity in head and neck squamous cell carcinoma (HNSCC) [22]. Our study uncovers another mechanism that nuclear SERPINB3 may enhance cell proliferation by inhibiting SENP3 and regulating NPM1 sumoylation in LUAD.

While our study establishes the nuclear localization and function of SERPINB3 in LUAD, the mechanism regulating its cytoplasmic-nuclear distribution remains unknown. Evidence from other cancers suggests that extrinsic stimuli like UV irradiation and chemotherapeutic drugs can induce SERPINB3 nuclear translocation [22, 23]. Therefore, elucidating the specific mechanisms governing this process in LUAD represents a critical area for future investigation.

Sumoylation is an important post-translational modification. It regulates protein localization, DNA replication and damage repair, genome stability, chromatin dynamics, phase separation, and ribosomal biogenesis [27, 28, 30, 46]. SENP3, as a de-sumoylation enzyme, plays critical roles in maintaining the homeostasis of sumoylation. NPM1, a major multifunctional nucleolar phosphoprotein, is one of the established substrates of SENP3. NPM1 has been implicated in hematopoietic and solid cancer development [47]. High expression of NPM1 is correlated with a lower overall survival in LUAD patients [48]. The Arf tumor suppressor binds NPM1 and enhances its sumoylation. While both major sumoylation sites of NPM1 (K230 and K263) can undergo SUMO modification [43], and their mutation has been shown to impair cell proliferation with the K263 mutation exhibiting more pronounced effects, the precise mechanisms by which SUMO-NPM1 promotes proliferation in lung cancer remain to be fully elucidated. Although previous studies suggested that sumoylation site mutations may alter NPM1 subcellular localization [43], our immunofluorescence analysis of PC9 cells stably expressing wild-type NPM1 or NPM1 mutants (K230R, K263R and K230/263R) revealed no discernible impact on nucleolar localization (Fig. S5F, S5G), which is consistent with another report [34]. This indicates that the proliferative effects of SUMO-NPM1 in LUAD may not be mediated through changes in its subcellular localization. Therefore, further investigation is required to comprehensively characterize how NPM1 sumoylation regulates LUAD cell proliferation. Given that NPM1 contains SUMO-interacting motifs (SIM) and sumoylation enhances binding to SIM-containing proteins, NPM1 sumoylation may facilitate two cooperative mechanisms: (1) enhanced self-assembly through reciprocal SIM-SUMO interactions between NPM1 molecules, and (2) increased recruitment of other SIM-containing effector proteins. It is possible that this multivalent interaction network could promote the formation of higher-order condensates that ultimately drive cell proliferation.

Structural characterization of the NPM1-SENP3 interaction yielded unexpected insights. While previous yeast two-hybrid data suggested that the N-terminal region of NPM1 interacts with SENP3 [34], our AlphaFold3 modeling and biochemical validation identify the C-terminal aromatic domain of NPM1 (amino acids 242-294) as critical for SENP3 binding. This discrepancy highlights the importance of orthogonal validation for protein interaction studies, particularly for oligomeric proteins like NPM1 that may present artifactual interactions in certain assay systems.

In summary, our study demonstrates that SERPINB3 functions as a novel regulator of the SUMOylation pathway in lung adenocarcinoma by directly binding and inhibiting the deSUMOylase SENP3. This interaction leads to the accumulation of SUMOylated NPM1, establishing a mechanistic link between SERPINB3-mediated SENP3 inhibition and enhanced proliferative capacity in lung cancer cells.

## Materials and methods

### Plasmids

LPC-Flag-BirA-SERPINB3 overexpression plasmid was constructed using standard molecular cloning techniques, with primer sequences provided in Supplemental Table S1. For SERPINB3 knockdown experiments, we employed shRNA constructs previously validated in reference [17]. Similarly, the LPC-Flag-SERPINB3 and LPC-Flag-Δ6 expression constructs were used according to previous publication [17]. The information for construction of LPC-HA-SENP3, LPC-HA-NPM1, LPC-His-SERPINB3, pSIN-Flag-NPM1, pSIN-Flag-NPM1 mutants and pSIN-Flag-ARF plasmids is listed in the Supplemental methods.

The bimolecular fluorescent complementary plasmids LPC-VN, LPC-VC, LPC-VN-Flag-SERPINB3, LPC-VC-HA-NPM1, LPC-VC-HA-SENP3, LPC-VN-HA-NPM1 were constructed with primers in the Supplementary table. The prokaryotic expression plasmid was obtained by double digestion of BamHI and HindIII using pET-28a (+) and finally recombined with the gene of interest. The primers used are shown in the Supplementary table.

### Antibodies

The following antibodies were used in the experiments: SERPINB3 antibody for WB (1:1,000, Thermofisher, cat. #PA5-81010); SERPINB3 antibody for IF (1:500, Thermofisher, #PA5-30164). SENP3 antibody for WB and IF (1:1,000, Cell Signaling Technology, #5591); SENP3 antibody for IF (1:50, Santa Cruz Biotechnology, #SC-133149). NPM1 antibody for WB and IF (1:1,000, Abcam, #ab10530; 1:1000, Cell Signaling Technology, #92825S); NPM1 antibody for IP (Proteintech, #60096-1-Ig). Flag antibody for IP (Sigma, # F1804); Flag antibody for WB (1:1,000, Proteintech, #20543-1-AP). HA antibody for WB (1:1,000, Cell Signaling Technology, #3724S). IgG antibody for IP (Abclonal, #AC005, #AC011). SUMO2 antibody for WB (1:1,000, Cell Signaling Technology, #4971S). RANGAP antibody for WB (1:1,000, Proteintech, #27405-1-AP). α-tubulin antibody for WB (1:1,000, Proteintech, #66031-1-Ig; #11224-1-AP); β-actin antibody for WB (1:1,000, Proteintech, #66009-1-Ig). Lamin antibody for WB (1:1,000, Abcam, #ab16048). TTF1 antibody for IHC (1:200, Thermofisher, #MA5-31938). Cytokeratin 5 antibody for IHC (1:2,000, Proteintech, #66727-1-Ig). Secondary antibody for WB: IRDye 800 CW Goat anti-Rabbit IgG (H + L) (#926-32211) and IRDye 680 RD Goat anti-Mouse IgG (H + L) (#926-68070) from LICORBio (NE, US).

### Cell lines

Lung cancer cell lines PC9 (RRID: CVCL_B260), H1975 (RRID: CVCL_1511), HCC4006 (CVCL_1269) were cultured in RPMI 1640 supplemented with 10% FBS (# FSP500, Excell Bio, China) and 1% penicillin and streptomycin (Meilunbio, China). Human normal lung epithelial cell line Beas2B (CRL-3588) and HEK293T (CRL-3216) cells were cultured in DMEM supplemented with 10% FBS and 1% penicillin and streptomycin. The cells were maintained in standard cell culture conditions at 37 °C and 5% CO_2_ in incubator (MCO-170, PHCbi, Japan). All cell lines were purchased from COBIOER, China or the Cell Bank of Chinese Academy of Sciences and authenticated based on STR genotyping. All cell lines were checked for absence of *Mycoplasma* by PCR within 6 months of experiments.

### Human LUAD samples

Human LUAD samples and non-tumor adjacent tissues were harvested from Department of Thoracic Surgery, Shanghai Pulmonary Hospital, Tongji University and Department of Oncology, Basic scientific research center, Longyan First Affiliated Hospital of Fujian Medical University. The experimental procedures were approved by the Research Ethics Committee of Tongji University and Fujian Medical University.

### Retroviral and lentiviral infection

PC9 stable cell lines overexpressing Flag-BirA-SERPINB3 or Flag-SERPINB3 were obtained using retroviral infection as previously described [17]. Briefly, three plasmid system (gene of interest, helper and VSVG at the ratio of 4:3:1) was used to generate viral particles after transfection into HEK 293T cells using Lipofectamine 2000 (# 11668019, Invitrogen, US). Filtered viral supernatant along with 10 mg/mL polybrene (#H9268, Sigma, Germany) was used to infect the target cells. For lentiviral infection, the above helper plasmid was substituted with $\triangle$R8.91 plasmid.

### Bio-ID

PC9 cells overexpressing Flag-BirA-SERPINB3 plasmid were treated with and without 50 μM biotin for 24 h. Cells were harvested with RIPA buffer (50 mM Tris (pH 7.4), 150 mM NaCl, 1% Triton X-100, 1% sodium deoxycholate, 0.1% SDS) containing Protease inhibitor and phosphatase inhibitor. Cell lysates were rotated at 4 °C for 10 min, lysed by ultrasound for 10 sec (30% intensity), 14000 rpm, and centrifuged for 10 min at 4 °C, the supernatant was collected. The protein concentration was measured and protein lysates for WB were prepared to verify the expression of SERPINB3. The Streptavidin-HRP antibody was probed to validate the successful addition of biotin. After confirmation of the addition of biotin, Dynabeads (MyOne Steptavadin C1; Invitrogen, US) was used to pull down the biotinylated proteins. Usually, 200 μL beads with 500 μg proteins were incubated overnight at 4 °C with rotation. Then the beads were collected and washed twice for 8 min at 25 °C in 1 ml wash buffer 1 (2% SDS in dH_2_O); followed by washing once with wash buffer 2 (20 mM Tris, pH 7.4, 500 mM NaCl, 1% Triton X-100, 1 mM EDTA, 1 mM EGTA) for 5 min; washed once with wash buffer 3 (20 mM Tris, pH 7.4, 300 mM NaCl, 1% Triton X-100, 1 mM EDTA, 1 mM EGTA) for 5 min. Finally washed once with wash buffer 4 (20 mM Tris, pH 7.4, 100 mM NaCl, 1% Triton X-100, 1 mM EDTA, 1 mM EGTA) for 3 min. Bound proteins were removed from the magnetic beads with 50 μL of SDS-sample buffer boiling at 98 °C for 5-10 min. Proteins eluted from the streptavidin beads could be visualized by silver staining or Coomassie blue staining, and harvested for Mass Spectrometry.

### Western blot

Western blot analysis was performed as described previously [49]. Briefly, cells were lysed in RIPA buffer (#MA0151, Meilunbio, China) containing 50 mmol/L Tris (pH 7.4), 150 mmol/L NaCl, 1% Triton X-100, 1% sodium deoxycholate, 0.1% SDS, EDTA, supplemented with protease and phosphatase inhibitors (#HY-K0010, MCE Chemicals, China). Protein (30 mg) was loaded onto SDS-polyacrylamide gels, transferred to NC membranes (#10600002, Cytiva, Sweden), incubated with antibodies and visualized by ODYSSEY CLx (LI-COR, US).

### siRNA

siRNA sequences were shown in the Supplemental Table S,1. When the cell density reached 60% of the 60 mm dish, fresh culture medium was added and transfection was performed according to the protocols of the manufacturer. Briefly, 3 μL siRNA and 6 μL Hieff Trans® siRNA/miRNA transfection reagent (#20806ES02, YEASEN, China) were used and the transfected cells were collected after 72 h.

### SENP activity assay

hSUMO2-AMC (#SI520) was purchased from Lifesensors (PA, US). The experiment was performed according to previous publication [42]. Briefly, cells were lysed in the SEM buffer: 0.25 M sucrose, 20 mM MOPS-KOH (pH 7.4), 1 mM EDTA-NaOH (pH 8.0). Immediately before use, SEM buffer was supplemented with Cocktail (1/1000), PMSF (1/200), DTT (1/1000). Briefly, the cell lysates were sonicated to dissociate the aggregates. The protein concentration of lysates was determined and the same amount of samples were prepared with SEM buffer. Cell lysates (15 μg total protein) were dispensed into the 96-well plate on ice. The assay master-mix was prepared: for one reaction 500 nM SUMO2-AMC substrate was used and adjusted with the activity assay buffer: 50 mM Tris-HCl (pH 7.5), 0.1 mg/mL BSA, 10 mM DTT to a total volume of 50 μL (including the volume of lysates). After adding the assay mix, the 96-well plate was quickly spinned down to collect liquids at the bottom of the plate and the fluorescence was measured using 380 nm as excitation and 460 nm as emission with SpectraMax i3x (Molecular Devices, US).

### Nuclear protein isolation

Briefly, 10 cm dishes of cells were lysed in 600 μL hypotonic buffer (20 mM Tris-HCl pH 7.4-7.6, 10 mM NaCl, 3 mM MgCl_2_), ice bathed for 15 min, 30 μL 10% NP-40 was added and vortexed at high speed for 10 s. The lysate was centrifuged at 4 °C 3000 rpm for 10 min. The supernatant was transferred into a new EP tube, and the cytosolic protein was obtained. The tips were used to aspirate the liquid around the pellet, which was the nuclear part. 100 μL of IP Buffer (50 mM Tris-HCl pH8.0, 150 mM NaCl, 1 mM EDTA, 0.5% NP-40) was added to suspend the precipitate and ice bathed for 30 min (during which the vortex shaked several times to promote lysis). The lysate was centrifuged at 4 °C, 14000 rpm for 30 min. The supernatant was transferred into a new EP tube, and the nuclear protein was obtained.

### Protein expression and purification

#### Day 1

The pET-28a (+)-SERPINB3 and pET-28a (+)-NPM1 plasmids were transformed into BL21(DE3) competent cells (#TSC-E01, Tsingke, China), and the pET-28a (+)-SENP3 plasmid was transformed into Rosetta (DE3) competent cells (#TSC-E04, Tsingke, China), and cultured overnight at 37 °C.

#### Day 2

The single colony was collected into 5 mL of LB medium with kanamycin (50 μg/ml), and incubated for 8 h. The bacterial solution was transferred to 200 mL LB medium with kanamycin (50 μg/ml) to expand the culture. 1 mL of bacterial solution was taken, centrifuged at 12000 rpm to collect the pellet, 300 μL of RIPA lysate was added and lysed with sonication.

#### Day 3

IPTG (#10902ES08, YEASEN, China) was added to a final concentration of 0.2 mM to induce protein expression for 4 h in 25 °C. After the induction, 1 mL of the bacterial solution was taken, the pellet was collected by centrifugation at 12000 rpm, 300 μL of RIPA lysate was added and lysed with sonication. The remaining approximately 200 ml of bacterial solution was lysed in 15 ml lysis buffer (pH 8.0, 50 mM NaH_2_PO_4_, 300 mM NaCl, 10 mM imidazole), sonicated on ice for 10 min, 10 s/10 s until the bacterial solution became clear and transparent. The bacterial solution was centrifuged and the supernatant was removed and stored at −80 °C. 20 μL of Ni-NTA resin (#30210, QIAGEN, Germany) was taken and centrifuged at 5000 rpm for 1 min to remove the supernatant, and resuspended with lysis buffer. The Ni-NTA resin was added into the protein solution and incubated with rotation at 4 °C for 1 h. The resin was washed 3 times with wash buffer (pH 8.0, 50 mM NaH_2_PO_4_, 300 mM NaCl, 20 mM imidazole). After the last washing, resin was washed with Elution buffer (pH 8.0 filter sterilized, 50 mM NaH_2_PO_4_, 300 mM NaCl, 250 mM imidazole), the supernatant was the purified protein.

### Co-immunoprecipitation (Co-IP)

#### Nuclear protein IP

Two dishes of 150 mm or 6 dishes of 100 mm cells were prepared to isolate nuclear protein. 5% of the nuclear protein was taken and prepared with a total volume of 50 μL protein loading solution as input. The remaining nuclear protein was divided into 2 groups, and the reaction system with a total volume of 480 μL was obtained by adding IP Buffer, 20 μL Protein A/G Magnetic Beads (#HY-K0202, MCE, China). 1 μL IgG was added into the control group, 1 μL primary antibody was added into the experimental group, and incubated overnight at 4 °C by rotation. Next day, the beads were washed 4 times with IP buffer (50 mM Tris-HCl pH 8.0, 150 mM NaCl, 1 mM EDTA, 0.5% NP-40). No more than 50 μL of 1×loading buffer was added and boiled at 98 °C for 10 min. The results were revealed by SDS-PAGE and WB.

#### Total protein IP

A dish of 100 mm cells was prepared to isolate total protein. Briefly, protein lysates, primary antibody and protein A/G magnetic beads (#HY-K0202, MCE, China) were incubated overnight at 4 °C with rotation and the beads were washed 4 times with IP buffer (50 mM Tris-HCl pH 8.0, 150 mM NaCl, 1 mM EDTA, 0.5% NP-40) the following day. 50 μL of 1×loading buffer was added and boiled at 98 °C for 10 min. The harvested samples were subjected to SDS-PAGE and WB for detection.

### Immunohistochemistry

The immunohistochemistry experiment was performed according to the protocol obtained from Cell Signaling Technology (CST, US) with minor revision. Briefly, after deparaffinization with histoclear (#G1128, Servicebio, China), the slides were sequentially hydrated with 100%, 95%, 80%, 70%, and 50% ethanol. Then the slides were immersed in 10 mM citric acid antigen retrieval solution (#G1202, Servicebio, China), boiled on low heat for 10 minutes, and naturally cooled for 30 minutes to achieve antigen retrieval. Then, the endogenous peroxidase was quenched with 3% H_2_O_2_ (#323381, Sigma, Germany). The slides were blocked with 10% goat serum, the primary antibody was incubated overnight 4 °C. After incubation with the secondary antibody, the slides were visualized using the DAB kit (#G1212, Servicebio, China). The nuclei were stained with hematoxylin. Finally, the slides were dehydrated by gradient ethanol and mounted with mineral oil. The slides were visualized under Keyence microscopy (BZ-X800, Keyence, Japan).

### Co-immunofluorecence

The co-immunofluorescence experiment was performed according to the protocol obtained from the Cell Signaling Technology (CST, US). Briefly, the cells were cultured in 24-well plates containing crawlers pieces until 50% density. Cells were washed once with 1×PBS, and fixed with 4% paraformaldehyde for 20 minutes. After washed with 1×PBS, cells were treated with Triton X for permeabilization. Then the cells were incubated with primary antibody and latterly the secondary antibodies. Finally, the fluorescent signal was visualized by Keyence microscopy (BZ-X800).

### Bimolecular fluorescence complementation assay (VN-VC)

When the cell density of the 6-well plate reached 60%, transfected according to the requirements of the manufacturer. 2 μL plasmid and 6 μL Lipofectamine™ 2000 Transfection Reagent (#11668019, Invitrogen, US) were used to transfect into cells, and visualized under Keyence microscopy (BZ-X800) after 36-48 h.

### Gel filtration chromatography

HEK293T cells were transfected with plasmids encoding Flag-SERPINB3, HA-NPM1, and His-SENP3 using PEI reagent (Polysciences, 23966) according to the manufacturer’s instructions. After 48 h, cells were collected and washed once with cold PBS buffer (137 mM NaCl, 2.7 mM KCl, 10 mM Na₂HPO₄, 2 mM KH₂PO₄), then lysed in 500 μL of 0.5% NP-40 buffer (20 mM Tris-HCl, pH 8.0, 140 mM NaCl, 5 mM KCl, 0.5% NP-40, 1 mM PMSF, 10 mM imidazole) supplemented with protease inhibitor cocktail (Roche). Cell lysates were incubated with Ni-NTA beads at 4 °C for 4 h with slow rotation, followed by washing with buffer (20 mM Tris-HCl, pH 8.0, 140 mM NaCl, 5 mM KCl, 0.1% NP-40, 1 mM PMSF, 10 mM imidazole). The enriched protein complex was eluted with 500 μL elution buffer (20 mM Tris-HCl, pH 8.0, 140 mM NaCl, 5 mM KCl, 0.1% NP-40, 1 mM PMSF, 300 mM imidazole) and loaded onto a Superose 6 Increase 10/300 column (GE Healthcare, 29091596) pre-equilibrated with low-salt buffer (20 mM Tris-HCl, pH 8.0, 150 mM NaCl, 1% glycerol).

Alternatively, for gel filtration chromatography of the SERPINB3-NPM1-SENP3 complex in cell lysates, 500 μL of lysate was directly loaded onto the Superose 6 Increase 10/300 column. Chromatography was performed using an ÄKTA Pure 25 system, and protein complexes were resolved and collected in 500 μL fractions. Twenty microliters from each fraction were separated on 4%-20% gradient polyacrylamide gels, and blots were probed with the indicated primary antibodies.

### Xenograft tumor formation

Male Athymic Balb/c nude mice at 5 to 6 weeks of age were purchased from Shanghai Model Organisms Center, Inc (Shanghai, China). For each PC9 xenograft tumor, 6 × 10^6^ PC9-LPC, SERPINB3 and ∆6 were suspended in 200 μL PBS and implanted subcutaneously into the upper flanks of mice, respectively. After tumor formation, tumor size was measured by calipers every 2-3 days. The volume (V) is calculated by the formula: V (mm^3^) = (L × W^2^)/2, where L and W represent tumor length and width, respectively. The measurements end when tumor volume reaches 1000 mm^3^. Tumors were dissected at the endpoints after mice euthanasia. All mouse experiments were performed in compliance with the Institutional Animal Care and Use Committee guidelines at Fudan university.

### Primer and oligonucleotide

All primers and oligonucleotide sequences are listed in Supplementary Table S,1.

### Statistical analysis

Statistical analysis for experimental data was performed using GraphPad Prism 8 software. Data were presented as mean ± SEM or SD as indicated. The differences between two groups were analyzed using a Student’s two-tailed *t* test. Survival curves were compared using a log-rank (Mantel-Cox) test. Two way ANOVA test was used for multiple group comparison. Statistical significance level was set at *P* < 0.05.

## Supplementary information

Supplemental Methods and Figures

Supplemental Table

Original WB

## Data Availability

The data supporting this study’s findings are available on request from the corresponding author.
