# Supplementary material for: SERPINB3 enhances NPM1 sumoylation via inhibiting SENP3’s activity and promotes lung tumorigenesis
Source: Cell Death Dis. 2025 Dec 24;17(1):133. doi: 10.1038/s41419-025-08347-9 (PMC12847774; doi:10.1038/s41419-025-08347-9)
Supplement: Supplementary file 1 — Supplemental Methods and Figures [file 41419_2025_8347_MOESM1_ESM.pdf]

Supplemental Methods

Plasmid construction

The LPC-HA-SENP3 construct was generated by double digestion of BamHI and XhoI using LPC vector and finally recombined with the HA-SENP3 using the recombinant enzyme (#NR005-01B, Novoprotein, China). The LPC-HA-NPM1 and mutant plasmids were constructed by double digestion of BamHI and HindIII using LPC and recombined with the HA-NPM1. The LPC-His-SERPINB3 plasmid was constructed by double digestion of BamHI and HindIII using LPC and recombined with the His-SERPINB3. The pSIN-Flag-NPM1, pSIN-Flag-NPM1 mutants and pSIN-Flag-ARF plasmids were constructed by double digestion of BamHI and HpaI using pSIN vector.

Supplemental Figures

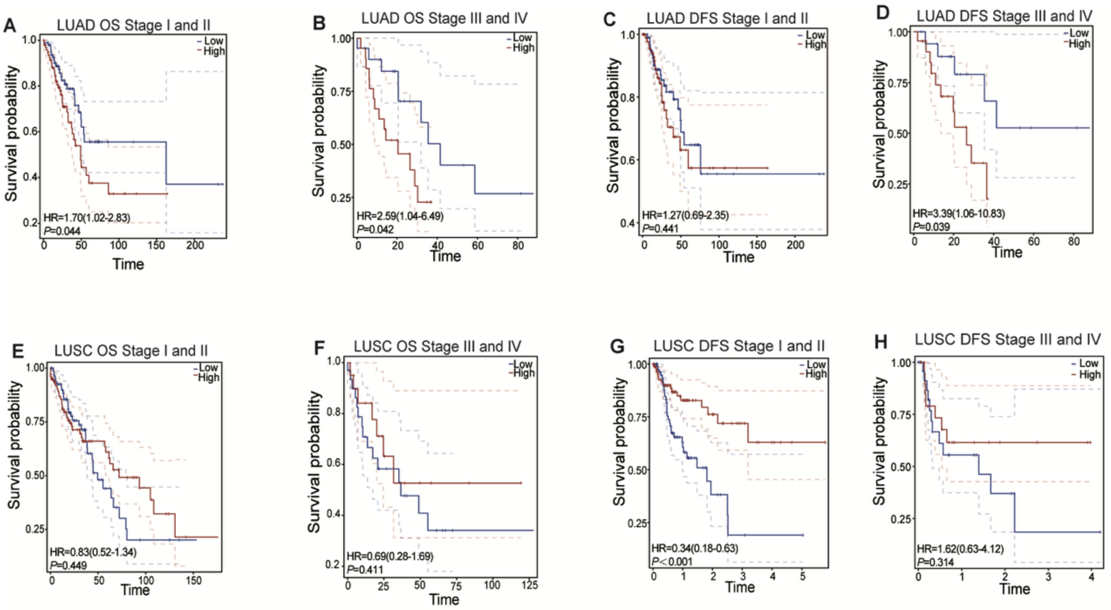

Figure S1: Kaplan-Meier survival analysis of SERPINB3 in human LUAD and LUSC samples. Supplemental to Figure 1.

(A and B) Kaplan-Meier survival curves of overall survival for stage I&II (A) and stage III&IV (B) LUAD patients from the TCGA cohort stratified by upper quartile (high) and lower quartile (low) of SERPINB3 expression levels. (C and D) Kaplan-Meier disease-free survival for stage I&II (C) and stage III&IV (D) LUAD patients from the TCGA cohort stratified by upper quartile (high) and lower quartile (low) of SERPINB3 expression levels. The statistical significance was determined by log-rank test. $*p < 0.05$. (E and F) Kaplan-Meier survival curves of overall survival for stage I&II (E) and stage III&IV (F) LUSC patients from the TCGA cohort stratified by upper quartile (high) and lower quartile (low) of SERPINB3 expression levels. (G and H) Kaplan-Meier disease-free survival for stage I&II ($***p < 0.001$) (G) and stage III and IV (H) LUSC patients from the TCGA cohort stratified by upper quartile (high) and lower quartile (low) of SERPINB3 expression levels. The statistical significance was determined by log-rank test.

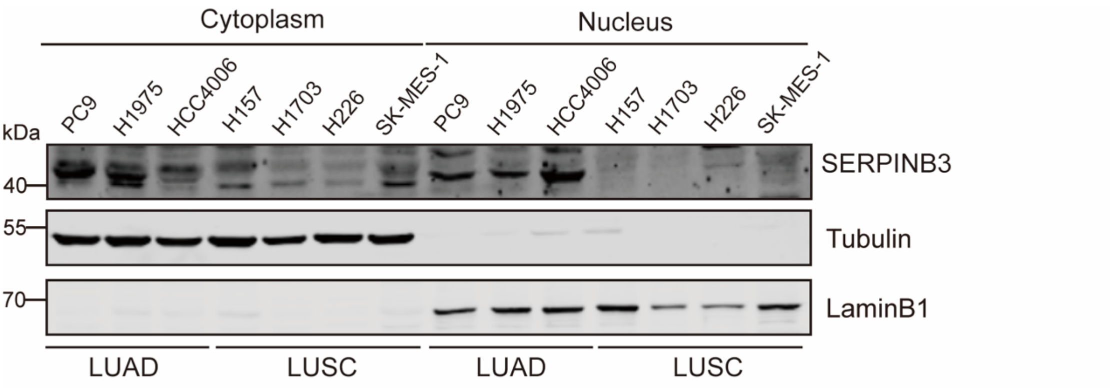

Figure S2 Western blot analysis of cytoplasmic and nuclear SERPINB3 in various LUAD and LUSC cell lines. Supplemental to Figure 2.

Western blot analysis of SERPINB3 from LUAD and LUSC cell lines' cytoplasmic and nuclear portion. Tubulin as a cytoplasmic protein marker, Lamin as a nuclear protein marker.

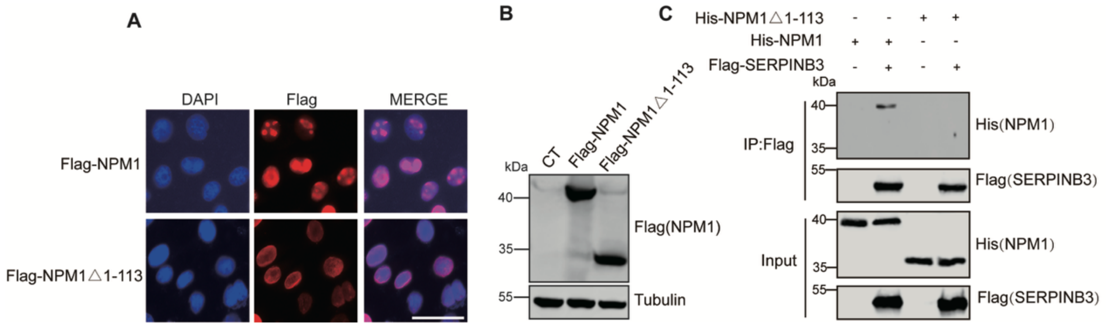

Figure S3: NPM1Δ1-113 fails to locate to the nucleolus

(A) Immunofluorescent analysis of Flag tag in PC9 cells overexpressing wild-type NPM1 and NPM1 Δ1-113. Scale bars: 50 μm. (B) Western blot analysis of Flag-NPM1 and Flag- NPM1 Δ1-113 in PC9 cells. (C) Western blot analysis of His-NPM1 and His-NPM1 Δ1-113 after co-IP using Flag antibody with purified proteins.

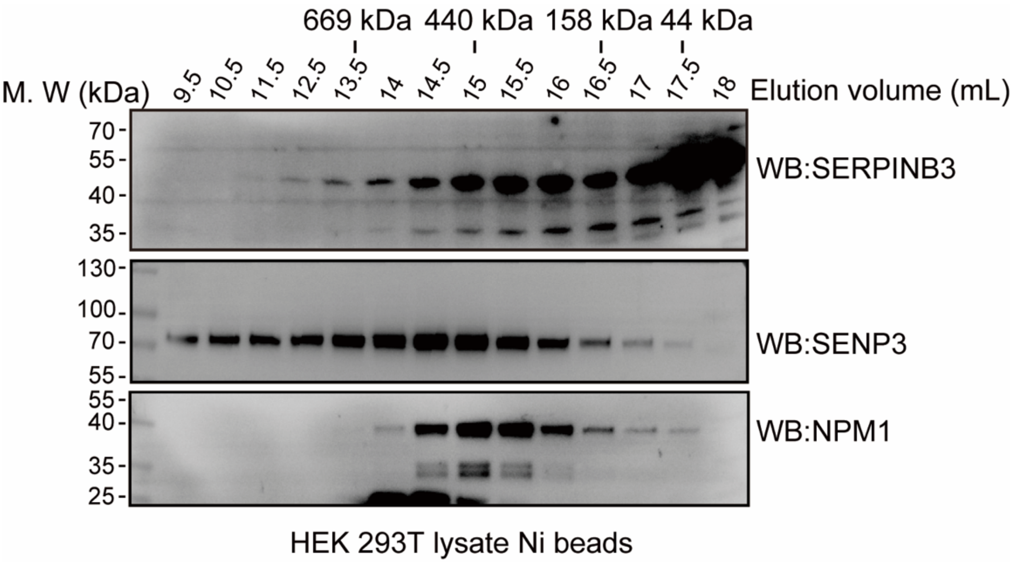

Figure S4 Gel filtration assay of HEK 293T lysate after Ni-NTA beads enrichment
The SERPINB3–NPM1–SENP3 complex enriched from HEK293 cells transfected with Flag-SERPINB3, HA-NPM1, and His-SENP3 was separated on a Superose 6 Increase 10/300 column. Fractions were resolved by SDS-PAGE and probed with the indicated antibodies.

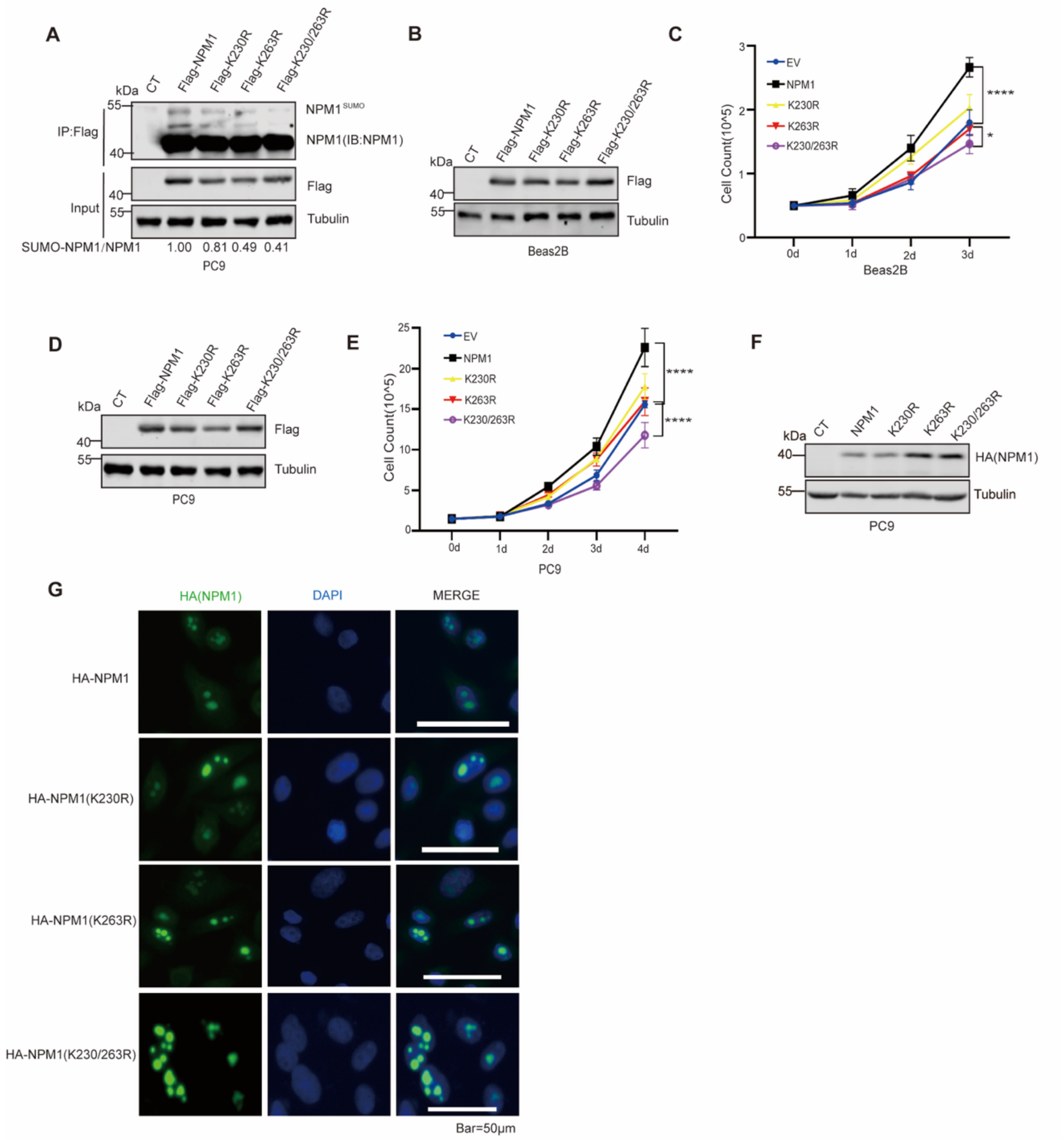

Figure S5 Proliferation is significantly impaired in the NPM1 K230/263R double mutant compared to the wild-type NPM1

(A)Western blot analysis of NPM1 and NPM1 mutants (K230R, K263R and K230/K263R) sumoylation in PC9 cells. The ratio of SUMO2-NPM1/tubulin was indicated. (B) Western blot analysis of NPM1 and NPM1 mutant (K230R, K263R and K230/K263R) expression in Beas2B cells. (C) Growth curve of Beas2B control, NPM1 and NPM1 mutants (K230R, K263R and K230/K263R) overexpression cells. Seed 5×10^4 Beas2B cells and count for 3 days. Values are mean ± SD from three independent experiments, ****$p < 0.0001$. *$p<0.05$. Two way ANOVA test was utilized for multiple group comparison. (D) Western blot analysis of NPM1 and NPM1 mutants (K230R, K263R and K230/K263R) expression in PC9 cells. (E) Growth curve of PC9 control,

NPM1 and NPM1 mutants (K230R, K263R and K230/K263R) overexpression cells. Seed 1.5×10^5 PC9 cells and count for 3 days. Values are mean ± SD from three independent experiments, $^{****}p < 0.0001$. Two way ANOVA test was utilized for multiple group comparison. (G) Representative images of immunofluorescent staining of HA tag, DAPI of PC9 cells expressing NPM1 and NPM1 mutants (K230R, K263R and K230/K263R). Scale bars: 50 μm.
