# Supplementary material for: SERPINB3 enhances NPM1 sumoylation via inhibiting SENP3’s activity and promotes lung tumorigenesis
Source: Cell Death Dis. 2025 Dec 24;17(1):133. doi: 10.1038/s41419-025-08347-9 (PMC12847774; doi:10.1038/s41419-025-08347-9)
Supplement: Supplementary file 3 — Original WB [file 41419_2025_8347_MOESM3_ESM.pdf]

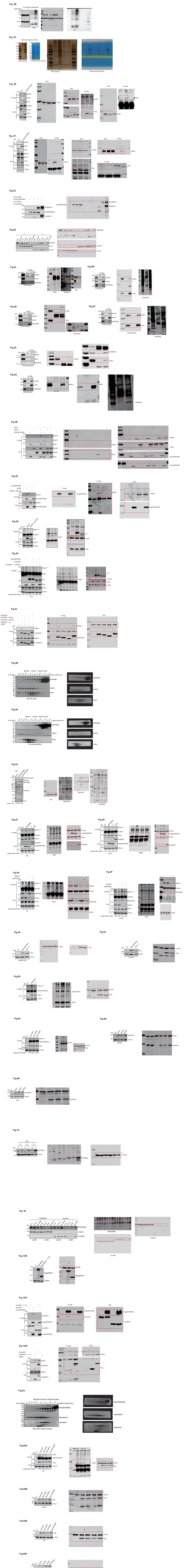
Fig. 2B
Fig. 2C
Fig. 2E
Fig. 2F
Fig.2H
Fig.2K
Fig.2L
Fig.2M
Fig.2N
Fig.2O
Fig.2P
Fig.2Q
Fig.3B
Fig.3E
Fig.3G
Fig.3H
Fig.3L
Fig.3M
Fig.3N
Fig.4A
Fig.4C
Fig.4D
Fig.4E
Fig.4F
Fig.5A
Fig.5C
Fig.5G
Fig.6A
Fig.6B
Fig.6D
Fig.7A
Fig. S2
Fig. S3B
Fig. S3C
Fig. S3D
Fig.S4
Fig.S5A
Fig.S5B
Fig.S5D
Fig.S5F
